# Effects of grazing intensity on soil labile organic carbon fractions in a desert steppe area in Inner Mongolia

**DOI:** 10.1186/2193-1801-2-S1-S1

**Published:** 2013-12-11

**Authors:** Jixin Cao, Xiaoping Wang, Xiangyang Sun, Lin Zhang, Yun Tian

**Affiliations:** Key Lab. Soil and Water Conservation and Desertification Combating, Ministry of Education, College of Soil and Water Conservation, Beijing Forestry University, Beijing, China; Beijing Forestry Carbon Administration (Beijing Forestry and Parks Department of International Cooperation), Beijing, China

**Keywords:** grazing intensity, soil labile organic carbon, desert steppe, land use

## Abstract

Grazing can cause changes in soil carbon (C) level. This study aimed to elucidate the response of soil labile organic carbon (SLOC) under four different grazing intensities: non grazing (NG), 0 sheep·ha^-1^; light grazing (LG), 0.91 sheep·ha^-1^; moderate grazing (MG), 1.82 sheep·ha^-1^, and heavy grazing (HG), 2.73 sheep·ha^-1^. Results showed that there was no significant difference in total soil organic carbon (TOC) and soil inorganic carbon (SIC) content from three soil depths (0-15 cm, 15-30 cm, and 30-45 cm) under different grazing intensities. However, the SLOC including particulate organic carbon (POC), light fraction organic carbon (LFOC), and readily oxidizable carbon (ROC) content at a depth of 0-15 cm decreased with the increasing grazing intensity among LG, MG and HG. The SLOC content at depths of 15-30 cm under the NG and LG were significantly higher than that under the MG and the HG. The TOC and SLOC content decreased with increasing depths of soil horizons, but SIC content increased. The variation trend of the density of different soil carbon fractions and the ratio of individual SLOC fractions to TOC were similar to that of the soil carbon content of corresponding fractions. These results indicated that MG and HG treatments caused C loss at 0-30 cm; and SLOC was more sensitive than TOC in response to different grazing intensities.

## Introduction

Grasslands of various types comprise approximately 29.4% of the global land [1]. Soil contains about 90% of total grassland systems carbon (C) [2], which store up to 30% of the world's belowground C [3]. Grazing, as one of the most important approaches, could modify the soil C stock in the grassland ecosystem and potentially influence climate change [4–7]. The investigation of the herbivores impact on the regulation of soil C is important to understand grassland ecosystems and to evaluate the contribution of grasslands to global C fluxes [8]. However, there are controversial reports on the effect of grazing on soil organic C level. Some studies reported that grazing increased soil C levels [2, 9, 10]. Other studies reported grazing had either no effect on [11] or decreased soil C levels [12–14]. The differences in soil C response to grazing may reflect differences in climate, inherent soil properties, landscape position, plant community composition and grazing management practices among reported studies [2].

Most of those previous studies focus on the effects of medium and long-term grazing on total soil organic carbon (TOC), while there are few for the soil labile organic carbon (SLOC) pool. Although TOC is an established indicator of soil quality, small changes in TOC resulting from changes in soil management are often difficult to measure, especially in a short-duration [15–18]. However, changes in small but relatively labile fractions of TOC may provide an early indication of soil degradation or improvement in response to management practices [16]. SLOC refers to carbon subject to intense impact of plants and microorganisms and characterized by its solubility, fast movement and ease of mineralization. Terms used to characterize SLOC in global soil science communities include, particulate organic carbon (POC), light fraction organic carbon (LFOC) readily oxidized carbon (ROC), soil microbial biomass carbon (SMBC) and dissolved organic carbon (DOC), etc [19, 20]. POC refers to the organic carbon which is combined with soil sand fractions (53~2000µm) and comprises primarily semi-decomposed plant residues [19, 21]. This fraction can be described as representing an intermediate pool with regards to decomposition [22]. Light fractions of soil is the fraction with a density less than 2.0 g cm^-2^, the carbon of which reflects a mixture of compounds that includes microbial biomass, partially degraded plant material and older, more humified, by-products of decomposition[23, 24]. ROC which is easily oxidized by potassium permanganate is considered to be a liable soil carbon pools [15, 16, 22, 25].

Inner Mongolia grassland located in the hinterland of Eurasia is the main body of temperate grasslands in northern China, accounting for about 22% of the total 400 million ha of grassland of China [26, 27]. 39% of Inner Mongolia grassland is desert steppe [13]. The object of our study is to investigate the effects of different grazing intensities on the soil carbon, especially on soil labile carbon in a desert steppe area in Inner Mongolia of China.

## Materials and methods

### Study site

The study site is located in Shi Dorbod Qi (County), Ulanqab City of Inner Mongolia autonomous region, China (latitude: 41°47′ N, longitude: 111°53′ E, elevation: 1450 m). It is characterized by a typically continental climate. According to long-term meteorological observation, the mean annual temperature is 3.4 °C. The three highest monthly mean temperature is 21.5, 24.0, 23.5 °C in June, July and August, respectively. The annual precipitation averages 280 mm, with about more than 80% received from May until September. Annual average total sunshine time is 3118 h, and the annual mean amount of evaporation is 2300 mm. The dominant plant species of the study site were *Stipa breviflora, Artemisia frigida* and *Cleistogenes songorica*, accompanied by *Convolvulus ammannii, Heteropappus altaicu, Neopallasia pectinata, Kochia prostrate, Caragana stenophylla* and *Leymus chinensis*. The soil of the study site was classified as light chestnut soil [28].

### Treatment

**Figure 1:**
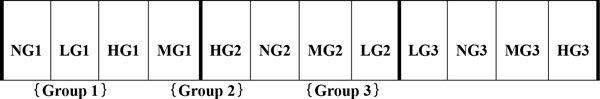
**Sketch map of different grazing intensities treatment plots**.

This steppe desert was where the grazing continued from 1988, when herdsmen began to settle down there to live, and due to the lack of effective management measurements, the steppe desert had begun to degradation before the enclosing. In June, 2004, about 50 ha relatively flat, uniform and native dessert steppe was enclosed for grazing. The fence grazing area was divided into three replicated groups by a complete randomized design (Figure 1). According to previous study results [29, 30], four 4.4 ha plots which mean four different grazing intensities (NG-non grazing 0, LG-light grazing 0.91, MG-moderate grazing 1.82, HG-heavy grazing 2.73 sheep·ha^-1^) were set up in each group. The local adult Mongolian sheep were the test ones. The period of grazing test was 6 months (June-November) every year from 2004 to 2007. Every morning test sheep were herded to the different sample plots, and every evening they were driven to the sheepfold during the grazing period.

### Soil sampling, laboratory and statistics analysis

Soil sampling was completed in the September of 2007. Twelve sites were randomly sampled in each plot with a soil auger. At each sampling site, soil samples were excavated at depths of 0-15, 15-30 and 30-45 cm. And then, soil samples of the same depth in each plot were mixed into a bulk sample [31]. For each soil sample, an accompanying soil bulk density sample was also taken at a near-by position by using a 100 cm^3^ soil ring.

Soil samples were air dried and passed through a 2 mm sieve. TOC content was determined by H_2_SO_4_-K_2_Cr_2_O_7_ pyrogenation method [32]. Soil inorganic carbon was determined by method described by following method [4, 32]: 5 g of < 2 mm (sieved), air dry soil sample was added to 25 mL of 3 mol·L^-1^ and then determined the volume of the released CO_2_ after being shaked for 4 min.

Particulate organic matter (POM) was separated from 2 mm soil following the method described by Burt [22]. 10 g of < 2 mm (sieved), air dry soil sample was weighed into a 125 mL Erlenmeyer flask, and then add 30 mL reverse osmosis deionized (RODI) water. Stopper sample and shake for 15 h at 200 oscillations·min^-1^. After that, soil suspension was rinsed by RODI water to sieve through 53µm mesh. The remains on the sieve were oven-dried at 110° C and weighed. The carbon content of POM was determined by the method of TOC analysis.

Light fraction (LF) organic carbon (LFOC) determination followed Gregorich and Ellert [23]: 25 g air-dry soil (<2 mm) was weighed into a 250 mL centrifuge tube and added with 50 mL NaI solution (1.7 g·cm^-3^). The mixture was shaken for 1 h at 200 rpm and then centrifuged for 20 min at 1000 rpm. Then the surface of NaI solution was filtered through a 0.45-µm nylon filter with aspirating. The LF which remained on the filter was washed with 75 mL CaCl_2_ solution followed by 75 mL distilled water to separate NaI from LF. Then LF was washed from filter to preweighed drying tins. After that, the tins were placed in the oven at 60°C to obtain the dry weight. Organic carbon in LF was also determined by H_2_SO_4_-K_2_Cr_2_O_7_ oxidation method.

ROC was determined by using 0.02 mol·L^-1^ KMnO_4_ oxidation method described by Weil et al. [16] and Burt [22]. Earlier studies [25, 33] suggested that 0.333 mol l·L^-1^ KMnO_4_ was a comfortable oxidizing agent. However, Weil, et al. [16] found that highly concentrated solutions (0.333 mol l·L^-1^) of KMnO_4_ were difficult to work with and tend to react with a large fraction of soil C which was not well distinguished from TOC and then improved the former method into a highly simplified method with 0.02 mol·L^-1^ KMnO_4._ Concrete procedures are as follows: stocking solutions of 0.02 mol·L^-1^ KMnO_4_ in 0.1 mol·L^-1^ CaCl_2_ and standard KMnO_4_ working solutions from 0 to 0.02 mol·L^-1^ were prepared. 5 g of < 2 mm (sieved), air dry soil sample was weighed into centrifuge tube. The soil samples were reacted with 20 mL the KMnO_4_ in 0.1 mol·L^-1^ CaCl_2_ solution. The centrifuge tube was shaken for 2 min and then allowed to settle for 10 min. 0.5 mL supernatant solution was diluted to 50 mL and absorbance was measured on a spectrophotometer which was set to read at 550 nm. The content of ROC was determined by the consumed KMnO_4_.

Soil carbon density refers to the carbon stock at specified depth in a unit area, and was calculated by multiplying carbon content of unit volume and the thickness of the given soil horizons [34, 35].

The data were analyzed by SPSS 17.0 with a one-way analysis of variance (ANOVA). The least significant difference (LSD) was performed to test differences when F values from ANOVA were significant (p < 0.05).

## Results and discussions

**Figure 2:**
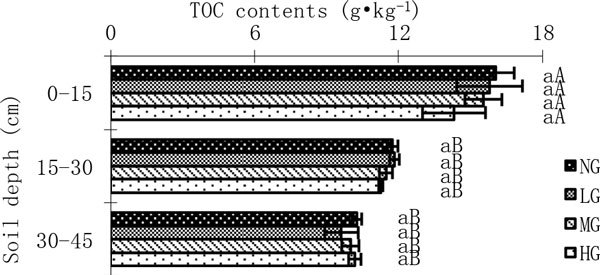
**TOC contents in different grazing intensity**.

**Figure 3:**
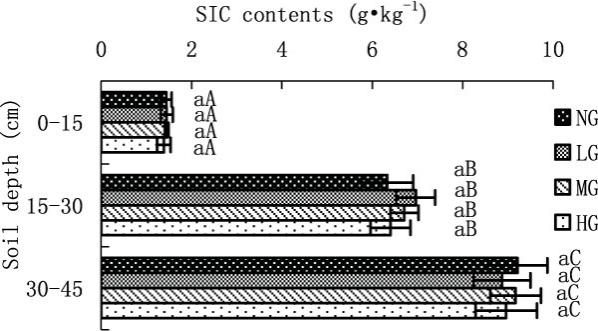
**SIC contents in different grazing intensity**.

**Figure 4:**
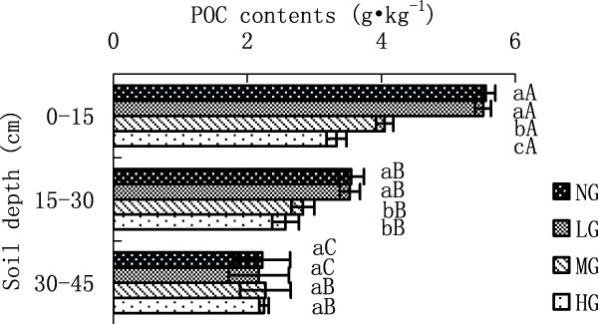
**POC contents in different grazing intensity**.

**Figure 5:**
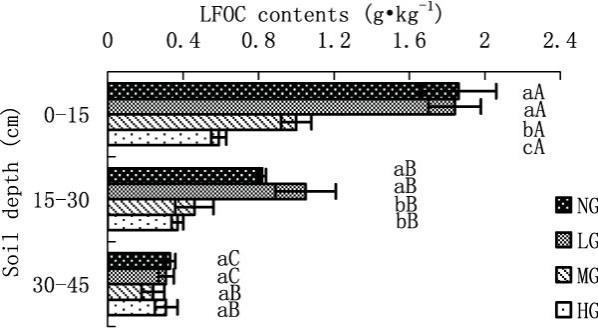
**LFOC content in different grazing intensity**.

**Figure 6:**
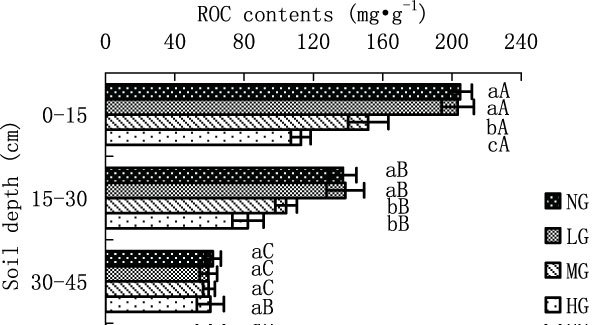
**ROC content in different grazing intensity**.

Figure 2 TOC contents in different grazing intensity. Statistics in Figure 2 are mean values with standard errors. Different small case letters indicate a significant difference with different grazing treatments (p < 0.05), and different upper case letters indicate a significant difference with different soil depth (p < 0.05). The same explanation also applies to Figures 3, 4, 5, and 6.

The content of TOC decreased with increasing grazing intensity at soil horizon of 0-15 cm (Figure 2). However, There was no significant difference of TOC content between different grazing treatments at all three depths (p > 0.05). TOC contents were the highest at the surface horizon (0-15 cm) and decreased with increasing soil depth. For all grazing treatments, TOC contents at the surface horizon were significantly higher than those at 15-30 cm (p < 0.05), which were higher than those at 30-45 cm with no significant differences (p > 0.05) (Figure 2).

SIC contents were not significantly different with increasing grazing intensity at all three depth (0-15 cm, 15-30 cm and 30-45 cm) (p > 0.05) (Figure.3). The variation trend of SIC contents with increasing soil depth was opposite to that of TOC. SIC contents of four grazing treatments were significant lower at the surface horizon than those at 15-30 cm, which in turn were also significant lower than those at horizon of 30-45 cm (p > 0.05) (Figure 3).

The soil organic carbon is determined by C input mainly from aboveground litter production, root turnover and animal excreta, and C output through soil respiration, erosion and leaching s4]. In our study, increasing grazing intensity did not cause significant variation for TOC contents at all three soil horizon. This may be explained by that TOC pool size and distribution reflects past carbon inputs and long-term accumulation process, and tends to respond slowly to management practices changes [15, 36, 37]. This result is consistent with the study result of Wang [11], but inconsistent with some other studies [2, 9, 10, 12–14]. This is mainly due to the differences of environment conditions and grazing time. Nevertheless, TOC was sensitive to overgrazing and slow to recover merely by enclosure when it declined following overgrazing in the semiarid typical steppes in Inner Mongolia [4].

Soil inorganic carbon is composed of lithogenic inorganic carbon and secondary carbonate [38], and most of SIC is in the form of calcium carbonate [39]. According to Pan's estimation [39], soil carbon stock of China was 110 Pg (1 Pg = 10^15^g), including 50 Pg TOC and 60 Pg SIC, which was mainly stored in Northwest and North China. Our study results show that after short-term grazing, SIC differed insignificantly with increasing grazing intensity at all three soil horizons. This result is consistent with Stavi's study [40]. However, Reeder et al. [41] found that SIC content increased after long-term heavy grazing compared with non-grazed treatment in short-grass steppes.

For all the grazing treatments, TOC contents decreased with increasing soil depths, while SIC contents increased. These results are consistent with Yang [42].

### SLOC content in different grazing intensities

#### *POC content in different grazing intensities*

POC contents tended to decrease with increasing grazing intensity at 0-15 cm (NG-5.56, LG-5.52, MG-4.05 and HG-3.33 g·kg^-1^) and 15-30 cm (NG-3.55, LG-3.53, MG-2.83 and HG-2.57 g·kg^-1^) soil horizon among LG, MG and HG (Figure 4). POC content in HG at 0-15 cm was significantly lower than that in MG, which in turn was significantly lower than that in LG and NG(p < 0.05). The content in LG was similar to that in NG at 0-15 cm. The POC content in HG at 15-30 cm was similar to that in MG, but significantly lower than other two gazing treatments (p < 0.05). Grazing did not cause significant differences at 30-45 cm with increasing intensity. The POC contents in NG and LG were significantly decreased with increasing soil depth of 0-45 cm (p < 0.05). The POC contents in MG and HG at surface horizon were significantly higher than that at 15-30 cm (p < 0.05), which were similar to those at 30-45 cm.

#### *LFOC content in different grazing intensity*

Grazing caused LFOC content in a significant decrease at surface horizon (NG-1.86, LG-1.84, MG-1.00 and HG-0.59 g·kg^-1^) among LG, MG and HG (p < 0.05), the LFOC content of which was the lowest (0.59 g·kg^-1^) (Figure 5). The LFOC content in LG at 0-15 cm was similar to that in NG, which was also significantly higher than that in MG and HG. The LFOC contents in NG and LG at 15-30 cm (NG-0.82, LG-1.05, MG-0.46 and HG-0.37 g·kg^-1^) were significantly higher than those in MG and HG (p < 0.05). There were not significant differences at 30-45 cm between different gazing treatments. The LFOC contents in NG and LG were significantly decreased with increasing soil depth of 0-45 cm (p < 0.05). The LFOC contents in MG and HG at surface horizon were significantly higher than that at 15-30 cm (p < 0.05), which were similar to that at 30-45 cm.

#### *ROC content in different grazing intensities*

ROC differed significantly at surface horizon (NG-204.72, LG-204.38, MG-151.65 and HG-112.74 mg·kg^-1^) among LG, MG and HG (p < 0.05), in which the ROC content was the lowest (Figure 6). The ROC content in LG was slightly lower than that in NG at surface horizon, but with no significant differences. At 15-30 cm, ROC contents (NG-137.00, LG-138.38, MG-104.46 and HG-82.22 mg·kg^-1^) in NG and LG were significantly higher than those in MG and HG (p < 0.05). There were no significant differences among different grazing intensities at 30-45 cm horizon. ROC contents in NG, LG and MG decreased significantly with increasing soil depth. ROC content of HG treatment at surface horizon was significantly higher than that at 15-30 cm, which was slightly higher than that at 30-45 cm, but with no significant differences.

While TOC did not change obviously, its labile component may decrease substantially under grazing [4]. After 22 years' grazing in *Leymus chinensis* steppe, ROC content (0.333 mol·L^-1^ KMnO_4_ oxidization) and SMBC content at 0-10 cm decreased by 22.0% and 27.9% respectively, while TOC content decreased insignificantly [18]. The imposition of a cattle grazing pressure is higher than the average for 6 and 8 year periods caused in a significant reduction in SMBC which decreased by 24% and 51%, while the TOC contents were similar [43]. We found similar trend in our study: at a soil depth of 0-15 cm, compared with LG treatment, POC, LFOC, and ROC contents of MG and HG treatments decreased by 27% and 40%, 46% and 68%, 25% and 45%, respectively. Therefore, these SLOC properties measured by different methods responded more rapidly to land management practices, and can provide a more sensitive measure of changes in the organic matter status of soils Than TOC [15, 16, 23, 43, 44].

In our results, we also found that TOC and SLOC contents at surface horizon were significantly higher than those at sub horizon (15-30 cm). This is due to that TOC is mainly from soil surface C accumulation including litter, roots, excrement and urine of animals in rangeland. Additionally, SLOC contents decreased more rapidly with increasing depth compared with TOC, especially in NG and LG treatments. This might be explained by that the different distribution pattern of various components of soil organic carbon in the soil profiles [45].

Statistics in Table 1 are mean values with standard errors in brackets. Different small case letters in a row mean significant different with different grazing treatment (p < 0.05), and different upper case letters in a row mean significant different with soil depth (p < 0.05). The same explanation applies to Table 2.

**Table 1:** Comparison of soil organic carbon density in different grazing intensity

Property	Depth (cm)	Treatment			
		**NG**	**LG**	**MG**	**HG**
TOC kg·^*m*-2^	0-15	3.22(0.18) aA	3.12(0.27) aA	3.17(0.20) aA	2.91(0.28) aA
	15-30	2.48(0.06) aB	2.41(0.05) aB	2.38(0.06)aB	2.31(0.03)aB
	30-45	2.18(0.01) aB	2.05(0.16) aB	2.12(0.11) aB	2.18(0.09) aB
SIC kg·^*m*-2^	0-15	0.29(0.02) aA	0.29(0.03) aA	0.30(0.01) aA	0.28(0.03) aA
	15-30	1.33(0.12) aB	1.42(0.08) aB	1.39(0.09) aB	1.31(0.09) aB
	30-45	1.96(0.13) aC	1.89(0.12) aC	1.79(0.24) a C	1.92(0.17) aC
POC kg·^*m*-2^	0-15	1.12(0.02) aA	1.09(0.01)aA	0.83(0.03)bA	0.68(0.04)cA
	15-30	0.75(0.03) aB	0.72(0.03) aB	0.58(0.02) bB	0.53(0.04) bB
	30-45	0.47(0.09) aC	0.46(0.09) aC	0.48(0.09) aB	0.48(0.02) aB
LFOC g·^*m*-2^	0-15	373.26 (29.94)aA	363.29 (30.84)aA	203.61 (14.34)bA	120.78 (11.53)cA
	15-30	173.27 (4.92)aB	214.01 (32.34)aB	95.85 (21.06)bB	75.99 (7.37)bB
	30-45	69.67 (7.63)aC	65.95 (8.50)aC	51.52 (13.66)aB	65.58 (13.21)aB
ROC g·^*m*-2^	0-15	41.04 (1.25)aA	40.11 (1.43)aA	30.99 (2.71)bA	22.94 (1.72)cA
	15-30	28.89 (2.14)aB	28.21 (2.12)aB	21.63 (1.41)bB	16.82 (1.68)bB
	30-45	13.25 (1.20)aC	12.67 (1.22)aC	12.59 (0.68)aC	12.99 (1.85)aB

**Table 2:** Rate of soil labile carbon to total organic carbon content in different grazing intensity (%)

Property	Depth (cm)	Treatment			
		**NG**	**LG**	**MG**	**HG**
POC/ TOC	0-15	34.91 (3.04)aA	35.52 (1.69)aA	26.11 (0.59)bA	23.79 (3.15)bA
	15-30	30.22 (1.55)aAB	29.83 (0.90)aAB	24.62 (1.26)bA	22.80 (1.95)bA
	30-45	21.65 (4.21)aB	22.71 (4.74)aB	22.48 (3.09)aA	22.12 (0.59)aA
LFOC/ TOC	0-15	11.75 (1.55)aA	11.93 (1.80)aA	6.45 (0.46bA	4.24 (0.59)bA
	15-30	7.00 (0.08)aB	8.93 (1.50)aA	4.00 (0.81)bB	3.28 (0.28)bA
	30-45	3.19 (0.37)aC	3.23 (0.41)aB	2.45 (0.67)aB	3.03 (0.64)aA
ROC/ TOC	0-15	1.29 (0.09)aA	1.31 (0.16)aA	0.98 (0.11)bA	0.80 (0.09)cA
	15-30	1.17 (0.07)aA	1.17 (0.10)aA	0.91 (0.04)bA	0.73 (0.09)bA
	30-45	0.61 (0.06)aB	0.62 (0.01)aB	0.60 (0.05)aB	0.59 (0.06)aA

### Soil organic carbon density

Table 1 shows that densities of POC, LFOC, and ROC decreased significantly with increasing grazing intensity among LG, MG and HG at surface horizon. Densities of three SLOC fractions in LG were similar to those in NG at surface horizon. At 0-30 cm soil depth, densities of POC, LFOC, and ROC in MG and HG accounted for 78% and 67%, 52% and 34%, 77% and 58% of corresponding density in LG, respectively. Densities of SLOC at 15-30 cm in NG and LG were significantly higher than those in MG and HG (p < 0.05). There were no significant differences for SLOC at 30-45 cm between different grazing treatments. Effects of grazing on densities of TOC and SIC were not significant at all three soil depths. The densities of TOC, POC, LFOC, and ROC decreased with increasing soil depth in all grazing treatments. However, SIC densities increased with increasing soil depth. These variation trends of different C fractions densities were similar to those of C contents of corresponding fractions.

Soil C density is decided by the contents of different soil C fractions, soil bulk density and contents of gravel. In our study site, there are little gravels in 0-45 cm depth soil. Therefore, soil C contents and bulk density are the main factor for calculating correspondingly soil C density. Our results showed that the variation trends of individual C fractions density were similar to those of correspondingly C contents with increasing grazing intensity. This is due to that soil bulk densities were insignificant differences with increasing grazing intensity at each soil horizons (data not shown). Some studies showed that the effects of herbivores trampling were significant, which caused increasing soil bulk density, especially in surface horizon [46–49]. However, other studies showed that grazing had no significant effects on soil bulk density [40, 43, 50]. The differences of these results may be due to the different inherent soil properties and grazing time.

### Ratio of Labile carbon

Table 2 shows that ratios of POC content to TOC content which varied from 34.91 to 23.79% at surface horizon in NG and LG treatments were significantly higher than those in MG and HG (p < 0.05). This trend of ratio was similar to that of ratio of LFOC content to TOC content which varied from 11.75 to 4.24% at surface horizon. Ratio of ROC content to TOC content varied from 1.29 to 0.80% at the surface horizon. For ratio of ROC content to TOC content, grazing caused significantly decreased at surface horizon with increasing intensity among LG, MG and HG. However, ratio of ROC content to TOC content in LG is similar to that in NG at surface horizon. At 15-30 cm, ratios of POC content to SOC content in NG and LG were significant higher than those in MG and HG (p < 0.05). This trend is similar to those for ratios of LFOC content to TOC content and ROC content to TOC content at 15-30 cm. At 30-45 cm, there were no significant differences between different grazing treatments for all ratio properties. All these ratio properties decreased with increasing soil depth.

The ratio of SLOC contents to TOC contents might be correlated with the difference in the distribution of plant root systems, the concentration of soil organic carbon, and the content of soil clay as well [^19]^. The relative changes in SLOC contents and TOC contents are more readily seen when they are expressed as a ratio of SLOC to TOC, which is lower for the heavy grazing [43]. Our results were consistent with this conclusion. Table 2 showed that the ratios of SLOC to TOC were all decreased with increasing grazing intensity among LG, MG and HG at 0-30 cm. The ratios had no significant changes at 30-45 cm, because of no significant differences of three SLOC fractions and TOC.

## Conclusions

There was no significant difference in TOC and SIC content from three soil depths (0-15 cm, 15-30 cm, and 30-45 cm) under different grazing intensities. However, the three SLOC fractions (POC, LFOC and ROC) content at a depth of 0-15 cm decreased with increasing grazing intensity among LG, MG and HG. SLOC fractions content of LG were similar to those of NG at 0-15 cm. Three SLOC fractions content under the NG and LG were significantly higher than under the MG and the HG at 15-30 cm, however, grazing caused no significant differences at 0-45 cm. The TOC and SLOC content decreased with increasing depths of soil horizons, but SIC content increased.

The variation trend of the density of different soil C fractions and the ratio of individual SLOC fractions to TOC were similar to those of the soil carbon content of corresponding fractions. Compared with NG and LG, HG and MG treatments caused the density of different SLOC fractions and the ratios of individual SLOC fractions to TOC significantly decreased at 0-30 cm. The density of different SLOC fractions and ratios of individual SLOC fractions to TOC decreased with increasing soil depths of soil horizons, however, SIC density increased.

Therefore, MG and HG treatments caused loss of soil organic carbon, and SLOC was more sensitive than TOC in response to different grazing intensities.
